# Germline based SARS-CoV-2 specific B cell repertoire motif identified with novel sequence based bioinformatic pipeline

**DOI:** 10.3389/fimmu.2026.1823750

**Published:** 2026-08-05

**Authors:** Daniel Fridman, Lena Israitel, Areen Shtewe, Uri Hershberg

**Affiliations:** Human Biology Section, School of Life Sciences, Faculty of Science, University of Haifa, Haifa, Israel

**Keywords:** antigen response, computational biology, COVID 19, immune repertoire, LPA, selection, sequence motif

## Abstract

Human antibody diversification, achieved through gene selection and somatic hypermutation (SHM), is critical for protecting against diverse pathogens. This study investigates whether specific immune responses possess distinct receptor sequence patterns that differentiate them from the general immune repertoire. Utilizing data from an anti-SARS-CoV-2 vaccination study, we analyzed two properties of SARS-CoV-2 specific memory B-cells and compared them to the background immune repertoire. Driven by somatic hypermutation (SHM), B cells exhibit a highly dynamic nature. Consequently, groups sharing a direct lineage from a common progenitor are defined as B-cell clones. First, we studied substitution survival - the number of clones to survive amino acid substitutions across the variable region of the B cell receptor (BCR). Second, we analyzed clonal amino acid trimer usage patterns across the BCR gene to gain insight into prevalent genomic motifs found in different immune sub-repertoires. We demonstrated that these two metrics can effectively cluster and distinguish SARS-CoV-2 specific B cell responses. Furthermore, we observed that SARS-CoV-2-specific B-cells show an increased tendency to utilize and conserve a specific CDR2 motif derived from the VH3–30 gene and its alleles. Beyond identifying a specific germline motif related to SARS-CoV-2-specific B-cells response, our findings demonstrate that our novel analysis pipeline can successfully identify signatures of specific immune responses. We therefore suggest that using the methods described here could be key for the study of the substrate of B-cell selection and protective immunity in other vaccine and pathogen responses.

## Introduction

One of the main goals of adaptive immune receptor repertoire (AIRR) research is the search for indicators of disease response. Discovering such indications of repertoire shifts in response to specific immune responses in B cell receptor (BCR) repertoires is not an easy task because of the hypervariability found in the antigen binding regions. Different individuals have different genetic backgrounds and somatic histories of immune responses leading to highly divergent immune repertoires. Each individual’s immune repertoire is unique – individuals, even identical twins, can be differentiated by their BCR repertoires (1), at the same time the form of the BCR is constrained (2) as are the permissible substitution patterns along the BCR sequence (3). Despite these contrasting phenomena, recent studies have demonstrated that numerous metrics can serve as indicators of specific responses, including V-gene somatic hypermutation (SHM) patterns (4–6), V-gene usage frequency (5, 7), and Complementarity-determining region 3 (CDR3) sequence convergence (8–10). These metrics have shown potential in identifying SARS-CoV-2 responses and, in some cases, even pathogenic severity (4, 6). A particularly interesting phenomenon observed in these studies is the tendency of the SARS-CoV-2 immune response to converge to specific motifs and responses, making such selected patterns a strong indicator of an immune response (5, 6, 8–10). As the most variable region of the BCR, the CDR3 is the primary driver of antigen specificity alongside the CDR1 and CDR2 regions (11). Consequently, existing research frequently employs CDR3 analysis to indicate disease states and identify specific response motifs at the clonal level (8–10). While the CDR3 is undoubtedly significant, other regions also contribute to BCR-antigen interaction (12). Additionally, the CDR3 inherent hypervariability and high mutation rates in addition to deletions and insertion in its sequence make the CDR3 the most challenging area for germline identification and somatic mutation analysis (13). Thus, in our study we chose to focus on the CDR1 and CDR2 regions, which are both variable and crucial for antigen specificity to identify clonal changes and mutant selection during specific immune response to SARS-CoV-2 spike protein, as shown in antigen structure studies (14, 15).

We here show a methodology that compares sub repertoires of antigen specific clones to nonspecific sub repertoires and identifies antigen specific localized patterns of selection. To equally focus on all parts of the BCR sequence and identify selection of specific motifs we used two complementary metrics: (1) Substitution survival, pattern of clonal survival following amino acid substitutions at specific sequence positions along the V gene.; and (2) trimer usage, trimer usage across clones (9, 16) of a given sub repertoire (in this case of B cells sorted as responding to the spike protein of SARS-CoV-2) found across individuals. Both substitution survival and trimer frequencies were directly derived from BCR sequence data and are easily calculated. We here combine both metrics to identify both patterns of selection and their outcome on the same sub-repertoires.

In the computational analysis of BCR repertoires, k-mer utilization serves as a highly effective technique for both feature extraction and statistical modeling. By breaking down the variable region into short stretches of k consecutive amino acids, highly diverse sequence datasets are transformed into robust, fixed-size abundance distributions (17, 18). K-mer analysis can be applied directly when evaluating mutational landscapes where decomposing the targeted sequence regions ensures that the resulting k-mer frequencies accurately reflect broad clonal evolution, and also as feature vectors for machine learning classification, for instance, distinguishing Crohn’s disease or celiac patients from healthy controls by identifying enriched trimers with specific bio-physicochemical properties (19, 20). We are here applying this accepted metric for motif detection alongside our more novel metric which can pinpoint positions of positive and negative selection to create a more complete image of the impact of selection to a specific antigen/disease.

An additional innovation of our approach is the use of domain-based Latent Personal Analysis (LPA), to compare the distributions of our two metrics. We have previously shown domain-based LPA is very useful for characterizing difference in immune repertoires (21, 22). LPA is particularly suited for this task as it was created to study multi-scale data and can be used to identify distinct clusters defined not only by abundance, which most methods distinguish, but also by significant under-representation of specific properties at the scale of those properties expected appearance. When we applied these two metrics and compared them by LPA analysis we identified in the SARS-CoV-2 specific sub repertoires a distinct pattern of overly stringent negative selection in the CDR2 region alongside an overabundance of a set of overlapping trimer motifs that combined to make the germline CDR2 region of the IGHV3-30, IGHV3-30-3, IGHV3-30–5 and/or IGHV3–33 gene and its alleles. These novel patterns we identified match existing evidence that in SARS-CoV-2 responses are germline in nature, due to the virus’s interferences with somatic hypermutation (SHM) (4).

## Methods

### Datasets

We performed our analysis on the BCR repertoires of 4 individuals post SARS-CoV-2 vaccination (23). Heavy chain DNA sequences were taken and bulk sequenced from sorted B cells taken from a cohort of 5 individuals, previously infected and recovered from SARS-CoV-2, following a vaccination and a booster vaccination 2 weeks after the first vaccine dose (23). Samples were taken from each individual at several timepoints (at vaccination, 2 weeks after the first dose and 1 week after the second dose/3 weeks after the first dose). Memory B cells in each sampled time-point were sorted into spike protein binding (SP) and non-spike protein binding (SN) (23). Only positions 28 through 104 (included) of the V gene of the heavy chain (by IMGT numbering (24)) were considered in our analysis. Positions prior to 28 were omitted due to their proximity to the primer binding site, a source of potential technical error in sequencing data. The analysis concluded at position 104 to exclude the highly variable CDR3 region, which begins at the subsequent position according to IMGT^®^ numbering (24). We removed one individual (individual 7 in the experiment’s numbering) due to its extreme under sampling (< 70 clones per time point on average for the SP sub-repertiores).

### Sequencing data quality control

Prior to annotation and analysis with the ImmuneDB pipeline (25, 26), raw sequences underwent quality control via the pRESTO toolkit (27) to filter out low-quality reads and ensuring data quality. Due to their varied and often low quality all positions before position 28 were removed from our analysis.

### Sequence processing

The ImmuneDB pipeline (25, 26) utilizes the Change-O toolkit (28) to match BCR sequences to their nearest heavy-chain germlines and annotate mutations. Clonal assignment was then performed using a widely accepted threshold (29, 30), where sequences sharing the same V(D)J germline assignments and at least 85% amino acid CDR3 sequence identity are grouped into the same clone.

### Clonal analysis

To focus our study on the selection patterns between clones, rather within clone on individual sequences level, we chose to analyze the properties of clones as our primary unit of analysis. First, for the substitution survival analysis, we examined the fraction of clones within a sub-repertoire (defined as clones from an individual at a specific time point with a defined antigen specificity) that survived a substitution at a certain amino acid position. For this step, we analyzed all clonal sequences derived from the sequencing results. Second, for the trimer analysis, we selected a representative sequence for each clone in one of two ways – (i) The most abundant sequence within each clone, which we chose so as to minimize the impact of outlier mutants in the clone; or (ii) The consensus sequence, in which to preserve the complete mutational profile of each clone, we mapped all mutations observed across the clone’s unique sequences onto its germline sequence.

### Selection pressure

Selection pressure was quantified using the BASELINe (31) (Bayesian Estimation of Antigen-driven Selection in Ig Sequences, used the default setting – human model) method, as implemented in the SHazaM R package (28) (part of the Immcantation framework), As input we used all sequences annotated for their clonal associations, as required for this analysis.

### Statistics and calculations

In all cases we used non-parametric tests. Comparing medians and performing Spearman correlation tests. Significance in all cases was determined at p<0.001. We minimized the number of tests we used so that at this quite stringent p value there was no need for multiple testing correction.

### Visualization

All visualizations were made with the Python module Matplotlib Pyplot (32).

### Substitution survival

To assess the capability of B-cells to undergo amino acid substitutions throughout the BCR variable region - and by extension, the positive selection and negative selection that together shape the population of clones that survive and proliferate - we calculated the fraction of clones possessing amino acid substitutions (considering all observed sequences per clone compared to their inferred germline) at each position of the heavy chain variable (V) region across all sequencing data. This analysis was performed separately for each sampled sub-repertoire (clones from an individual, at a certain time point, with a specific antigen-specificity label), resulting in a vector of amino acid substitution fractions per sub-repertoire.

### Unique trimers

To study short localized signatures of diversification and selection, while maintaining high sequence resolution (9, 14), unique trimer AA frequencies in each repertoire were calculated from the representative sequence for each unique clone (see Above), converted into a series of overlapping trimers via a sliding window across the sequence. Each trimer was indexed by the position of its first amino acid. Trimers did not include gaps while preserving the IMGT amino acid indexing. Following (33), we count serine separately if it is encoded by the codons TCN or AGY. Serine is unique in that its two disjoint codon sets cannot be connected by a single point mutation leading them to exhibit distinct substitution patterns and evolutionary selection pressures (33). Treating them as separate entities prevents the masking of these distinct selection pathways, resulting in a potential of 21³ unique trimers in our analysis.

### Diversity

We employed the diversity (3) metric of order 1 (q=1) to characterize the predominant patterns within the population. Unlike richness, which counts all instances, over emphasizing very rare events order 1 diversity weights instances without bias to their abundance, removing very rare events. In this study, we employed the diversity calculation on trimer usage of clones belonging to a sub-repertoire.

(1) $$1D_{w,p}\equiv \exp(-\sum_{i=1}^{R_{w,p}}Pi_{w,p}\times \ln(Pi_{w,p}))$$

Equation 1*– Diversity estimates at q=1* (34)*. Pi represents the proportional frequency of the selected attribute across the sub-repertoire. Depending on the analysis, in our case, Pi denotes the frequency of a specific trimer across all clones.*

### Domain based latent personal analysis and data analysis

1. Latent Personal Analysis (LPA) – LPA was designed to identify the differences in documents based on the unique signature of their word distribution (22). We have previously modified LPA to differentiate between tissues by analyzing their clonal populations (21). We further modified it here to compare sub-repertoires and identify unique immunological signatures within antibody sequences data. Specifically, we analyzed substitution survival and trimer usage patterns (see above) in the variable region of the heavy chain across individuals under different sample conditions. This method allowed us to pinpoint unique patterns in specific samples to gain insight into the selection and diversification properties exhibited during specific immune response. Diversity and selection in B cell populations is based on both clonal shift (the selection of specific clones) and clonal drift (the selection of specific mutants in a clone). The former can be better characterized by looking at changes in V(D)J gene usage and the later by identifying specific somatic mutants and sets of mutations, but changes in repertoires are always a combination of both. Our analysis is to some extent agnostic to which of these two phenomena is the main source of selection as we look for specific patterns that are overly abundant or missing in one sub-repertoire compared to the expected when looking across sub repertoires.
2. LPA Data Preprocessing – Effective use of the LPA algorithm requires a clear definition of the study’s structural hierarchy: the domain, entities, and elements (22). The domain serves as the overarching dataset containing all sub-repertoire entities, while the elements represent the specific metric frequencies that define each entity’s profile. In our research these 3 components of LPA analysis were defined as follows:
• Entity: A sub-repertoire, consisting of all B cell receptor sequences derived from all samples in a specific subject at a given time point with a particular antibody specificity.
• Element: The term “element” refers to the fundamental unit of quantification within the LPA. Its specific definition is context-dependent, as the algorithm was applied to two distinct metrics: (1) Number of clones that survived a substitution mutation at a given amino acid position. (2) The frequency of a unique trimer (three-amino acid motif) identified within a specific sub-repertoire.
• Domain: The domain represents the overall distribution of (1) the substitutions per position or (2) unique trimers across all individuals, prior to its classification into specific sub repertoires.
3. Kullback-Leibler Divergence (KLDe) Distance (22) – KLDe distance is a metric used by the LPA algorithm to quantify how much a specific entity’s distribution (e.g., the substitution survivability or trimer frequency in a specific sub-repertoire) diverges from the distribution of the entire domain (e.g., the combined information of all the data). A positive large distance indicates a surplus of that element in a specific sub-repertoire(entity) and a negative distance indicates shortage/lack compared to the domain.
4. Principal Component Analysis (PCA) (35) – The LPA analysis produces a KLDe “signature” dataframe for each sub-repertoire (entity) with a dimensionality, corresponding to the number of element types analysis (No. amino acid positions or No. of unique trimers in the research described here). To reduce dimensionality and identify key differences between sub repertoire types we performed a PCA analysis of signature differences.
*5. trimers Preprocessing* – To better understand the properties of the trimers, two dataframes were created:
a. trimers_diversity – output of the analysis performed on each of the positions between 28 and 104 of the variable regions of the heavy chain, calculates the diversity of the trimers at each position for each sub-repertoire.
b. trimers_presence – a dataframe that shows in what positions and sub-repertoire each trimer is found.

6. *Median Comparison –* Following the LPA, the median distance from the domain score for each trimer, across individuals and time points, was calculated separately for the SP and SN sub-repertoires. trimers ranking in the top and bottom quartiles (i.e., those with the highest and lowest median distances from the domain) were selected for further analysis. These selected trimers were then used to filter the trimers_presence and trimer_diversity tables to investigate their specific properties.

### Data filtration

Due to insufficient amount of data in some cases, we occasionally encountered sub-repertoires with low clonal counts that could compromise the accuracy of our reporting. To mitigate this challenge, we filtered subject 7 and remained with 4 subjects (3, 4, 5, 6), as subject 7 contained comparatively small number of unique clones (see **Supplementary Table S1**).

## Results

### SARS-COV-2 vaccination specific immune repertoires exhibit specific selection patterns that differ than those of a combined non-specific repertoire

**Figure 1:**
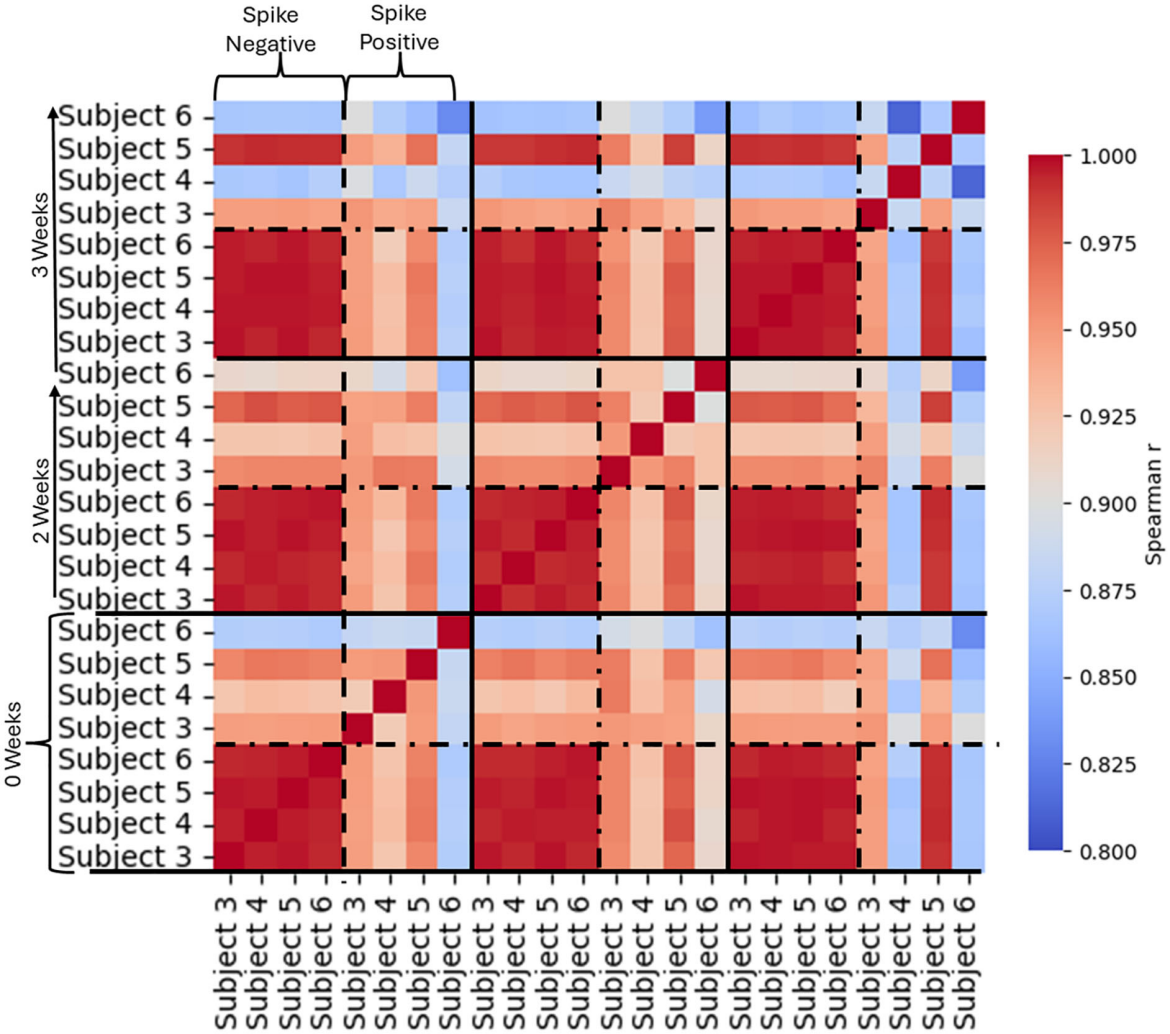
Heatmaps of the Spearman-r correlation between the frequencies of clones with substitutions at amino acid positions 28–104 in the heavy chain variable region of the human BCR. The presented correlation of by position substitution patterns are all statistically significant (P < 0.001). The correlations are shown between each sub-repertoire divided by time point and antibody specificity. The sub-repertoires are organized by time-point, antibody specificity and subject. Solid lines separate between different time points; 0 weeks, 2 weeks and 3 weeks, and dashed line between different antibody specificity types; SN and SP.

The frequency of clones found with substitutions at specific V gene positions is highly correlated between different individuals (36). We hypothesized that this high level of correlation represented the effects of negative selection (3) and could be decreased if we compared repertoires with high levels of positive selection. We therefore looked at the patterns of per position substitution survival in 4 individuals who had undergone spike protein specific vaccination to SARS-CoV-2. The immune repertoires of these individuals were divided into sub-repertoires based on antigen specificity (specific SARS-CoV-2 - SP vs. nonspecific SN), and sampling time point (pre-vaccination, two weeks post-first dose, and one week post-second dose/three weeks post first dose), trimer. When the entire repertoires of these individuals were compared, they showed the expected near perfect Spearman correlation of per position amino acid substitution survival across clones (>0.99 in all cases). When we considered the non-specific SN repertoires, they were similarly also very highly correlated across timepoints and individuals. However, the SARS-CoV-2 specific SP repertoires, in contrast, exhibited lower correlation, both internally and with the SN sub-repertoires. Furthermore, this internal correlation among the SP repertoires progressively decreased over the vaccination timeline (**Figure 1**).

### SP sub-repertoires exhibit unique substitution survival patterns with an excess of negative selection at CDR2 and CDR2 adjacent positions

**Figure 2:**
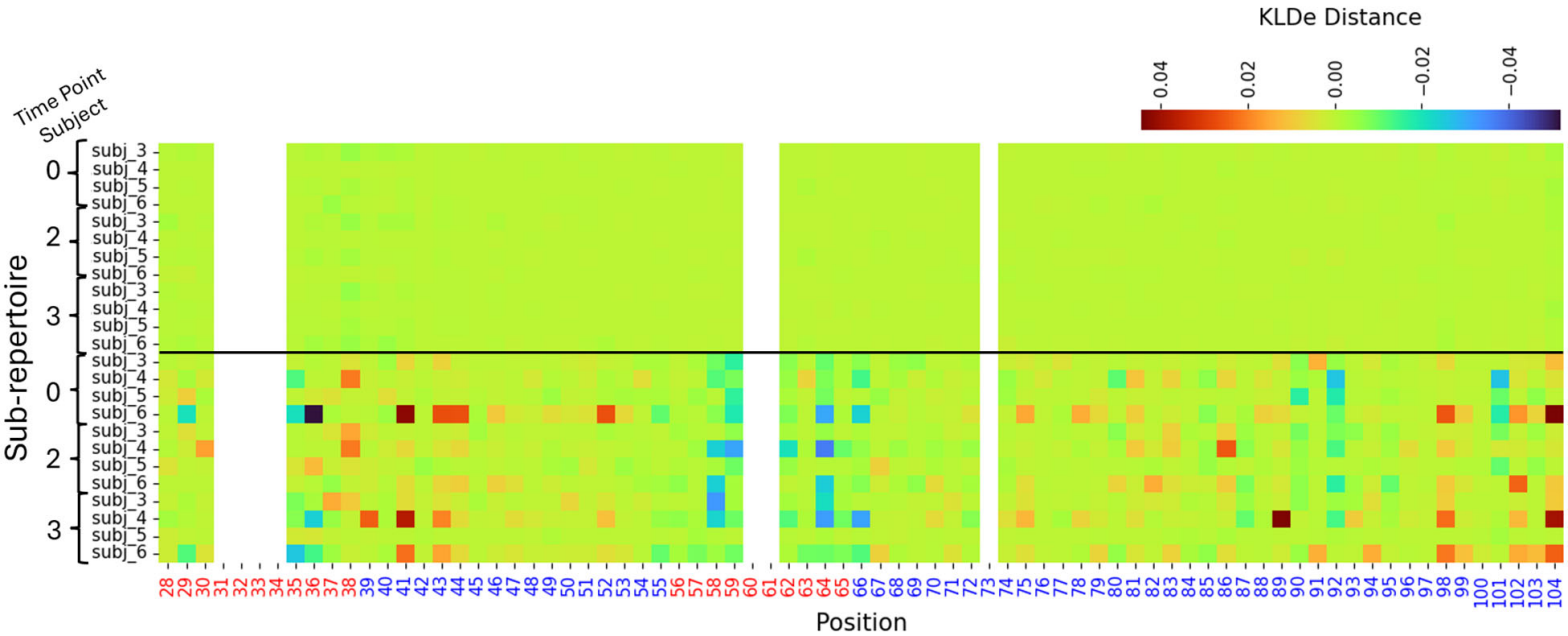
Heatmap showing the KLDe of each repertoire relative to the entire LPA domain, based on substitution survival across AAs 28–104 of the BCR heavy chain region from the full dataset. CDR positions are marked in red. Each line represents a sub repertoire that corresponds to a subset of clones derived from a single subject at a specific time point, with defined specificity toward the SARS-CoV-2 spike protein (SP or SN). Green squares represent positions at which a sub repertoire does not differ from the overall repertoire (the domain). Blue squares are cold spots that exhibit less clonal survival (i.e. more negative selection) than expected while red squares are hotspots that exhibit more clonal survival (i.e. more positive selection) than expected. Positions which are gapped to maintain IMGT numbering are marked as blank spaces.

To determine the cause of the lower correlation within the SP sub-repertoires, we performed Domain-based LPA using the substitution survival patterns as input (see **Methods**). This analysis revealed that the SN sub-repertoires displayed uniform proximity to the domain, indicating similar patterns of by position substitution survival following in different SN sub-repertoires. In contrast, the SP sub-repertoires exhibited significant variability, characterized by distinct “hotspots”, positions where the fraction of clones surviving a substitution were substantially higher and “coldspots”, positions where survival post substitutions was lower than the trends of the entire repertoire (**Figure 2**).

**Figure 3:**
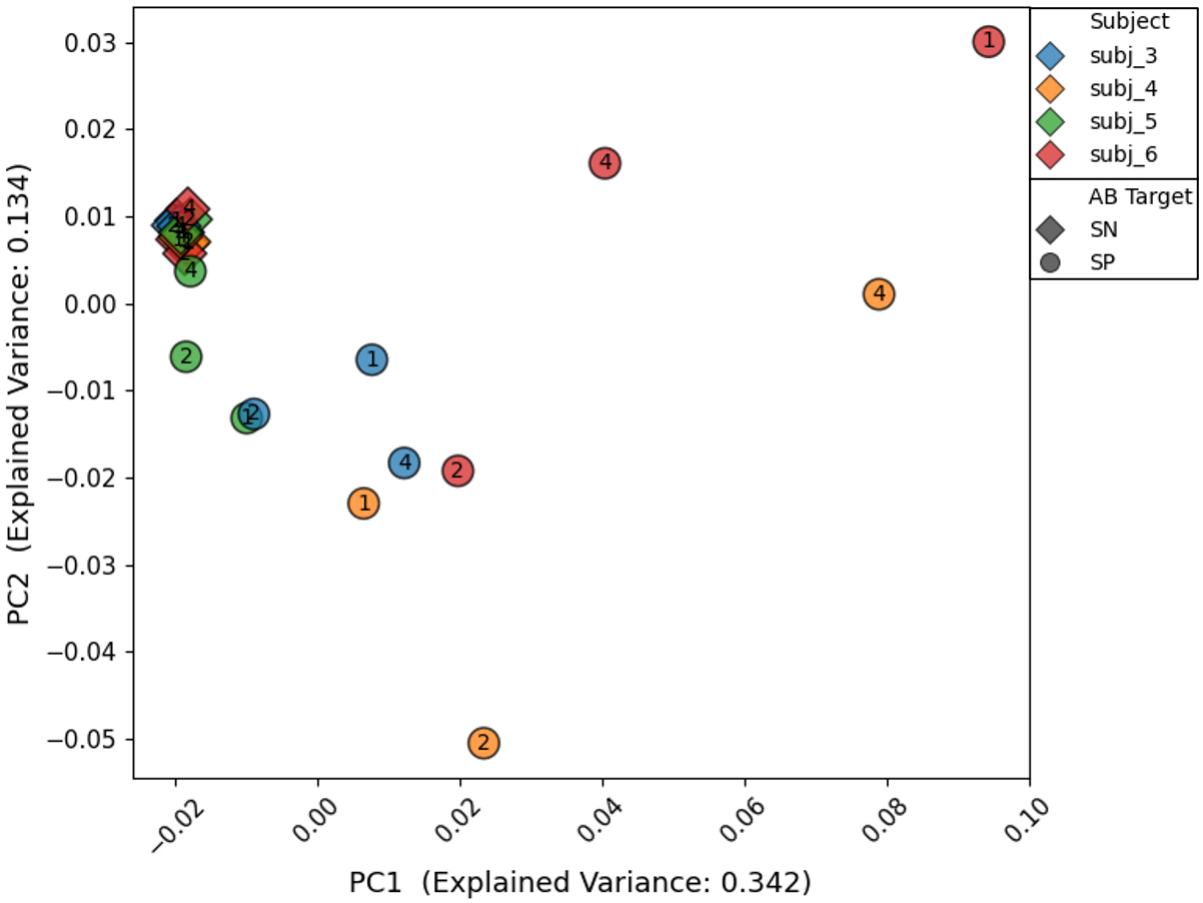
First two dimensions of PCA of sub-repertoire KLDe distances from the domain based on substitution survival measures. Circles represent SP sub-repertoires and diamonds SN sub-repertoires. Data points are color-coded by subject, with time points labeled within each symbol (1- pre vaccination, 2–2 weeks before first dose, 4- 3weeks after first dose and 1 week after second dose).

SP sub-repertoires show noticeable substitution pattern differences at certain amino acid positions while SN do not (**Figure 2**), Interestingly, the CDR2 region of SP sub-repertoires (positions 58-67) is less tolerant of substitutions, showing lower survival rates following amino acid substitutions compared to other regions. To check the overall combined effect of these differences, we performed a PCA on the KLDe distances calculated for each sub-repertoire (**Figure 3**). The scatter results demonstrate that the SARS-CoV-2 (SP) specific sub-repositories diverge from the history of immune responses, thereby forming two distinct clusters (**Figure 3**). Additionally, we here see, as we did above, that SN repertoires are similar at different time points while the SP sub repertoire continues to change with every boost. No further sub-clustering was observed when the features were visualized by different PC combinations (**Supplementary Figure 1**).

To pinpoint the main V gene positions that cause the difference between SP and SN substitution survival patterns we calculated a median KLDe distance for each antibody specificity sub-repertoires derived from each subject. The analysis reveals two key observations. First, within the SP sub-repertoires, the positions with the greatest positive median distance (more survival at these position in SP clones compared to the domain) are predominantly located in the framework regions (FWR), while the positions with the greatest negative distance (less survival at these positions in SP clones compared to the domain) are in CDR2 or in positions adjacent to CDR2. Second, the SP repertoires overall exhibit a greater divergence when compared to the SN repertoires, all of whose median differences are more or less at zero (**Supplementary Figure 2**).

### SP repertoires differ from SN repertoires in their amino acid trimer motifs

**Figure 4:**
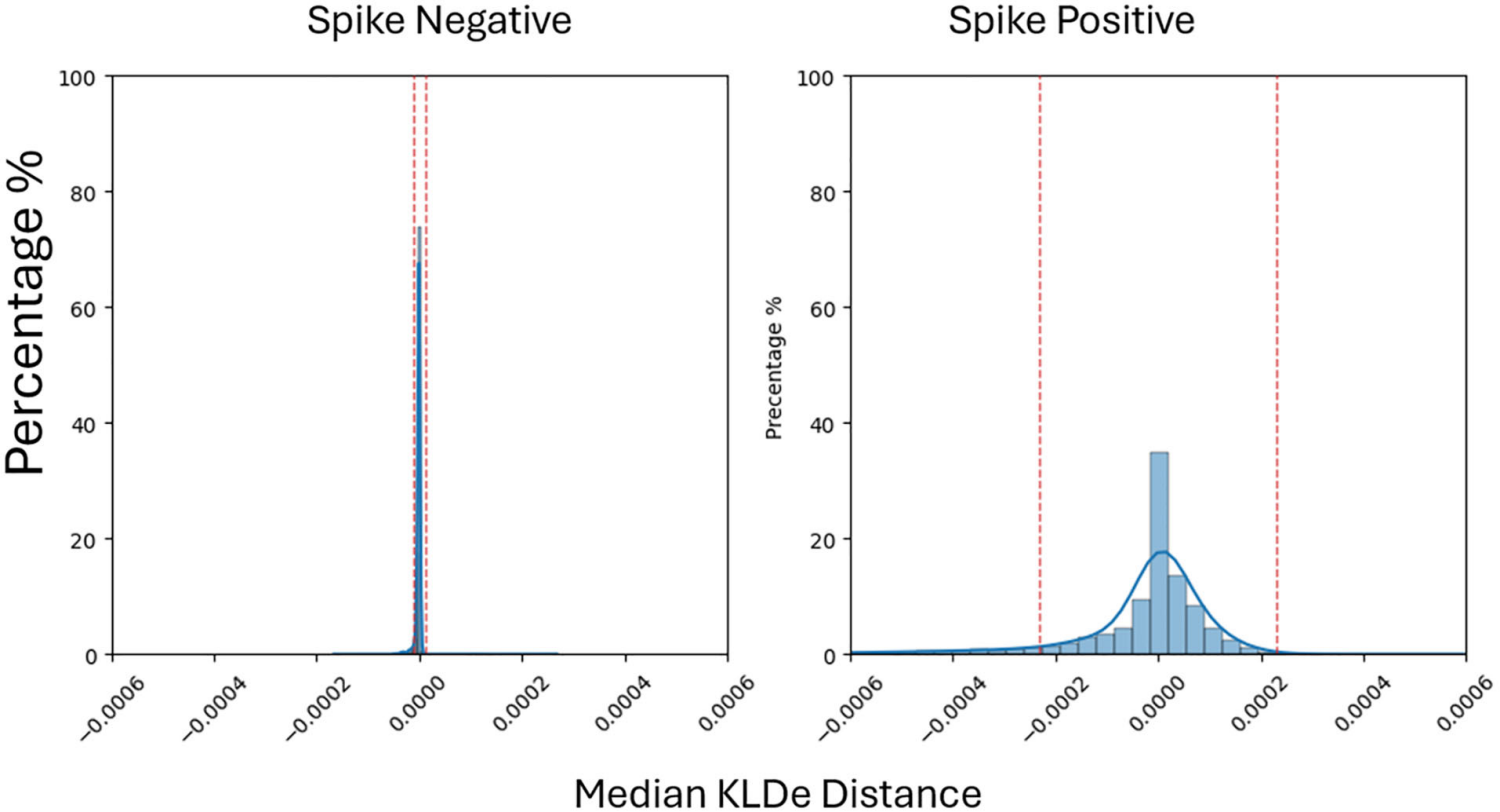
Histograms of the median KLDe LPA distance (for trimers calculated at diversity of order 1) per unique trimer for the SN (left) and SP (right) sub-repertoires. Red lines indicate two standard deviations from the mean of the distribution.

**Figure 5:**
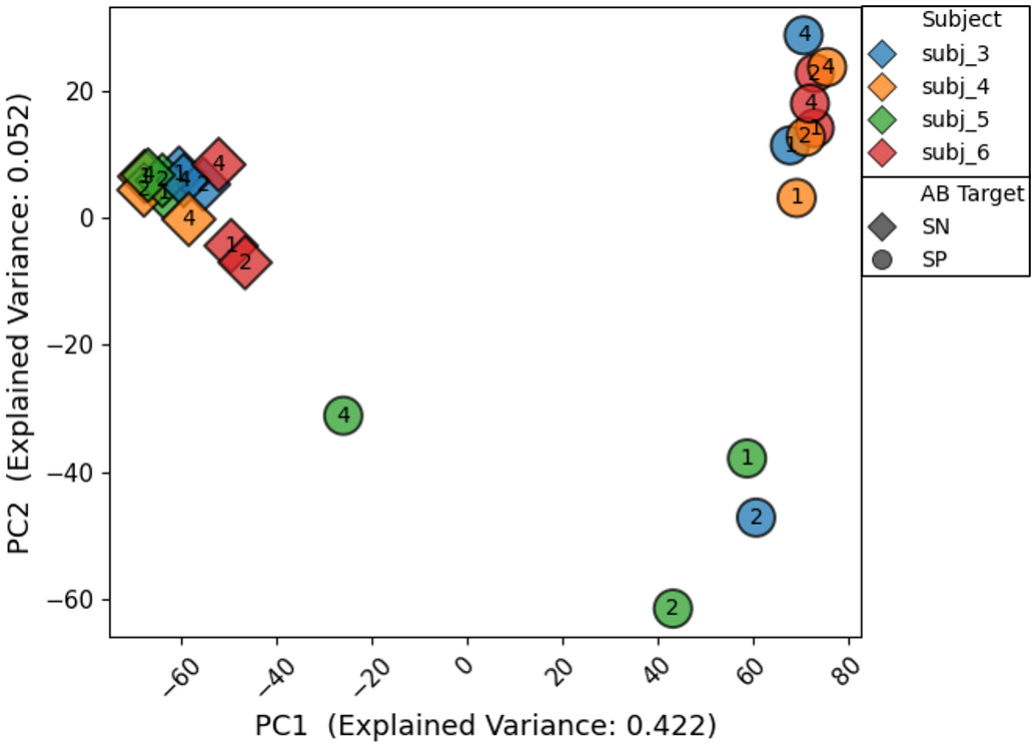
Scatter plot of PC1 and PC2 combination derived from PCA of repertoire KLDe distances relative to the entire domain which obtained from the trimer occurrences analysis. Circles represent SP sub repertoires and diamonds SN sub repertoires. Data points are color-coded by subject, as in **Figure 3**, with time points labeled within each symbol (1- pre vaccination, 2–2 weeks before first dose, 4- 3weeks after first dose and 1 week after second dose).

Having identified that patterns of substitution change in the response to the spike protein we next wanted to see if could identify specific amino acid motifs in the SARS-CoV-2 vaccination response. To do so we quantified the occurrences of each unique trimer across all sub-repertoires. Using Domain based LPA we now identified the distinct trimers usage in the SP sub-repertoires when compared to the shared domain (see Methods). As with the substitution patterns we here too found that in general the SN repertoires mostly had trimer patterns very close to the domain while the SP repertoires had more divergent trimer patterns (**Figure 4**). Using PCA to reduce the dimensionality of our comparison of each repertoire’s signature of difference from the domain, we once again found that even with just first two principal components, it was clear to see that the SN and SP sub-repertoires formed distinct clusters based on their antibody specificity (**Figure 5**). This clustering indicates that sub-repertoires with the same specificity share similar trimer usage profiles, while no distinct sub-clustering was observed while considering additional PC (**Supplementary Figure 3**).

### SP sub-repertoires consistently share only 9 trimer motifs, all in CDR2

**Figure 6:**
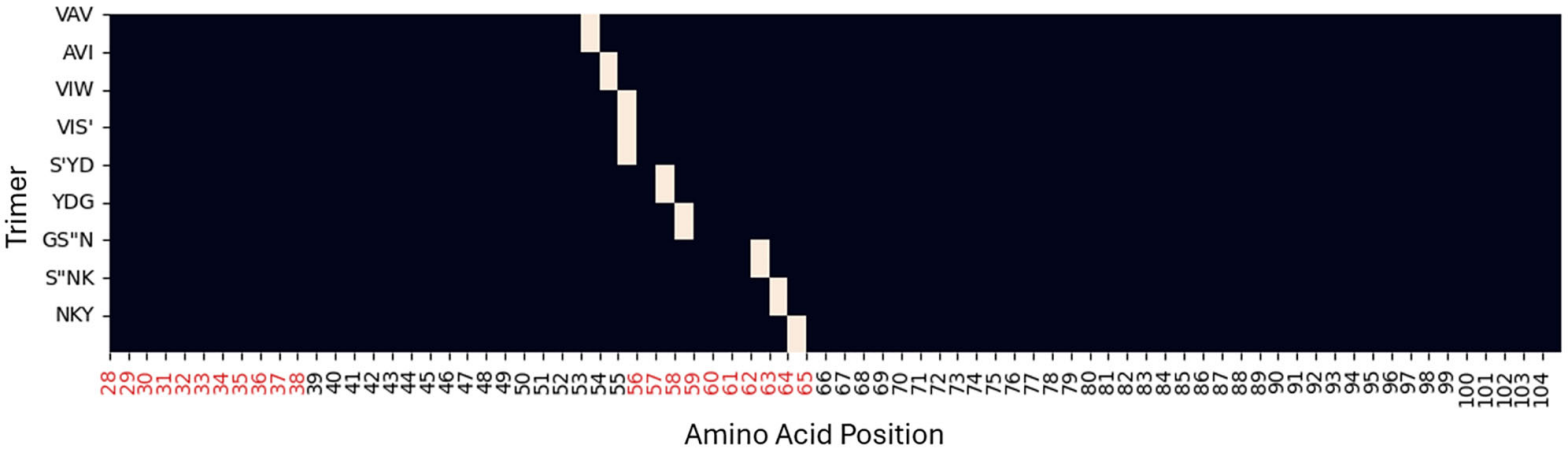
Heatmap of unique trimer occurrences overabundant in SP repertoires based on KLDe domain distance median score per individual and organized by the trimers’ first IMGT heavy chain sequence position. CDR positions are highlighted in red. Only trimers overabundant in at least 2 individuals (in the top quartile of overabundance) were noted. Cell color reflects trimer prevalence: beige-white color denotes presence in 4 individuals, while black indicates total absence.

To identify which trimer motifs were driving the differences between SP and SN observed in the PCA we calculated the by position order 1 diversity of trimers and kept only those trimers that contributed to the diversity of trimers in at least one position, in at least one sub repertoire. In this way we were left with 3,891 trimers to compare. We then calculated the median KLD of these trimers across the SP and SN sub repertoires and kept only those who were in the top quartile of over abundant trimers in at least two people. Only 9 (in which 2 are overlapping at position 55 - `VIW` and `VIS`) trimers were found in SP repertoires to be in the top quartile in terms of median distance from the domain across all 4 individuals, no others were found in even just two individuals. More interestingly, all 9 over abundant trimers formed a contiguous 12-mer across CDR2 and adjacent positions (**Figure 6**), note that due to 2 “gaps” in the complete overlap found trimers (lack of `ISY` and `DGS` at positions 56 and 59–61 accordingly) we receive sequence of 12 amino acids from 8 trimers rather than 10 (**Supplementary Figure 4**). This contiguous sequence of trimers formed the sequences ‘VAVISYDGSNKY’ and ‘VAVIWYDGSNKY’, which are germline sequences found in three specific V gene variants: IGHV3-30, IGHV3-30-3, IGHV3-30-5 (37, 38) and IGHV3-33. Interestingly these germlines are known to be overexpressed in responses to SARS-Cov-2 (39). To ensure that selecting the most abundant sequence per clone did not omit motifs present in less expanded sequences further away from the germline root, we repeated the analysis using the consensus sequences (which contain all mutations exhibited by a given clone - see **Methods**). This approach continued to demonstrate a distinct separation between the SP and SN clusters based on the PCA analysis (**Supplementary Figure 5**). Furthermore, we recovered a subset of 7 of the trimers identified in the initial analysis (which together still encode for the full CDR2 germline motif – *VAVISYDGSNKY)* and yielded no novel motifs (**Supplementary Figure 6**). Analyzing the SN trimers again representing each clone with its most abundant sequence, we identified a substantially higher number of motifs across the V gene, but with no clear set of motifs describing any part of the sequence and especially in the CDR region which shows multiple representative trimers at each position (**Supplementary Figure 7**), reflecting the diverse history of responses that makes up the SN sub-repertoires. Notably, we found no common trimers between the SN and SP results. Analyzing the SN trimers when each clone is represented by its consensus sequence raises even further the diversity of trimers from within and between individuals (as expected since the consensus sequence can be more mutated than the most abundant sequence but never less mutated). Due to this heightened diversity only a single trimer is found to be overly abundant in more than two individuals. Regardless of the type of consensus sequence used for this analysis it is important to remember that all trimers found to be in the top quartile of median overabundance in SN sub repertoires are actually quite close in their expression to that in the domain and show over abundancies far lower than those of the top quartile of the SP trimers (**Figure 4**).

### IGHV3-30, IGHV3-30-3, IGHV3-30–5 and IGHV3–33 with germline CDR2s are over abundant in SP clones

**Figure 7:**
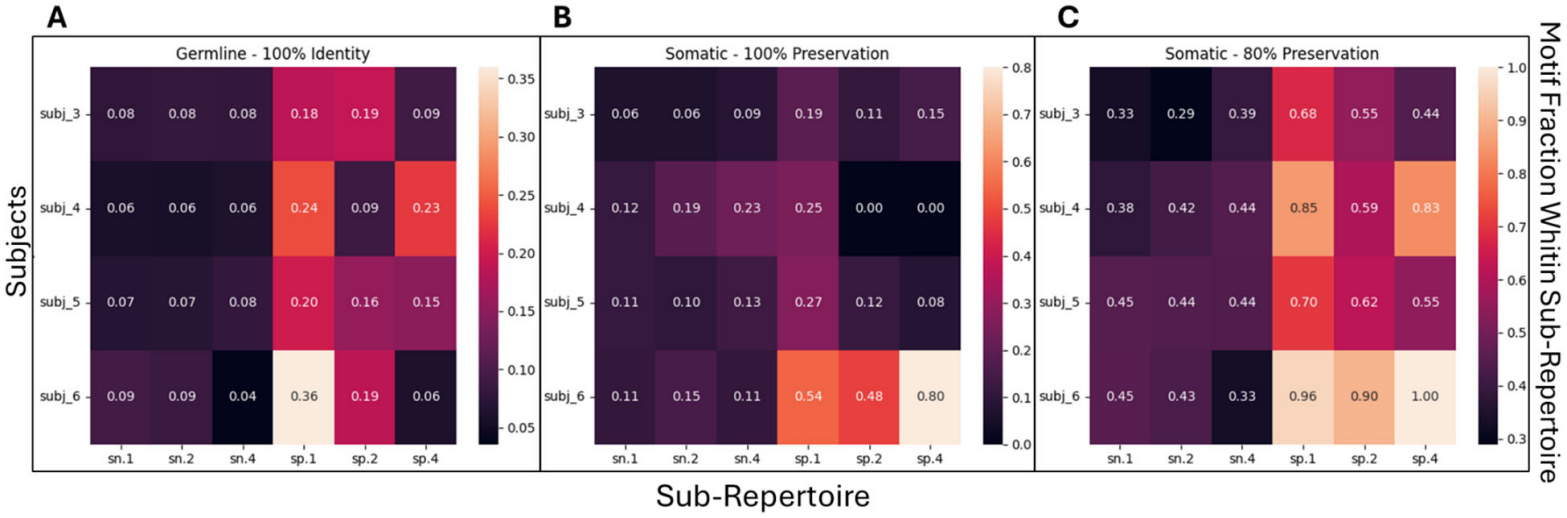
Sum of the frequencies of the ‘VAVISYDGSNKY’ and ‘VAVIWYDGSNKY’ motifs. **(A)** fraction of clones that perfectly matches either motif in their germline sequence. **(B)** fraction of clones with either motif in their germline sequence whose clonal variants have 100% identity to the motif sequence. **(C)** fraction of clones with either motif sequence in their germline sequence whose mutant variants have at least 80% sequence identity to the motif sequence. Cell color indicates the fraction of clones possessing either germline identity **(A)**, complete somatic identity **(B)** or at least 80% sequence similarity to the motif **(C)**; higher color intensity corresponds to a greater fraction.

Given that the motif we found was so clearly a germline sequence we wanted to see if we could more generally verify a differential impact of selection in the CDR2 of SP repertoires compared to SN repertoires. We did this in two ways:

**(1)** Focusing on the germline level, we scanned all clones featuring the ‘*VAVISYDGSNKY’* and ‘VAVIWYDGSNKY’ motifs, which is characteristic of the IGHV3-30, IGHV3-30-3, IGHV3-30–5 and IGHV3–33 alleles (37, 38). All individuals had these germlines in their repertoires but In the SN sub-repertoires, the motif’s germline frequency was consistently low (0.05-0.1). In the SP sub repertoires by contrast it was quite high at the first time point (0.2 -0.35) and went down (although still higher than in SN repertoires) in subsequent time points (0.1-0.2) (**Figure 7A**). To assess to what extent the observed germline motif was just the result of germline usage bias or reflected also selection bias, we checked to what extent the germline sequence was maintained during somatic development. We found that while differences in 100% conservation were only reliably observed at the firs time point (SP - 0.19-0.54; SN - 0.06-0.12), (**Figure 7B**), a >0.8 identity to the ‘VAVISYDGSNK’ or ‘VAVIWYDGSNKY’ motifs was found at much higher levels in SP clones than SN clones throughout (SP - 0.44-1; SN - 0.29-0.44), (**Figure 7C**). This goes hand in hand with our observation of more stringent negative selection in CDR2 from our localized substitution pattern analysis.

**Figure 8:**
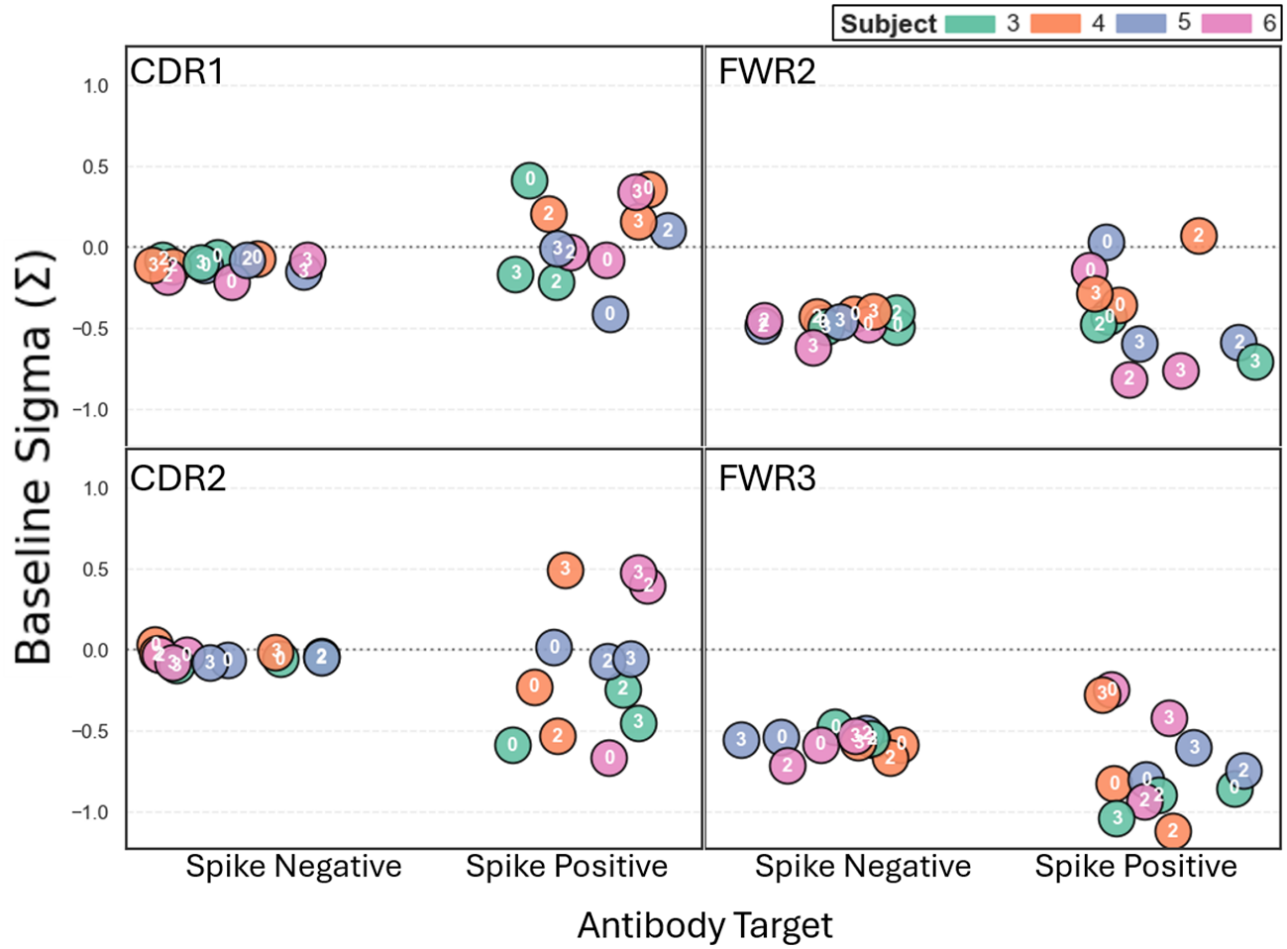
Baseline selection strength (Σ) across Variable Heavy (VH) regions for SP and SN repertoires. Analysis is of combined repertoires across individuals divided into three distinct timepoints following the first vaccination dose: 0 weeks (labeled as 0), 2 weeks (labeled as 2), and 3 weeks (labeled as 3). Panels display selection estimates for CDR (CDR1, CDR2) and FWR (FWR2, FWR3). The dashed horizontal line at y=0 represents neutral selection, with values below zero indicating purifying negative selection and those above positive selection.

(2) Finally, we compared the overall selection pressure (28, 31) in all FWR and CDR regions of the SP and SN clones, and found that while FWRs show some negative selection in both SN and SP repertoires while only SP clones show positive selection in CDR1 but negative selection in CDR2 (**Figure 8**).

## Discussion

We have shown here a novel method of detecting differential selection patterns and their outcomes when comparing antigen specific sub-repertoires to the nonspecific repertoires. This novel method identifies position specific substitution pattern differences and the underlying amino acid motifs that they create. We applied these methods to identify unique selection patterns in SARS-Cov-2 specific B cells, generated post SARS-Cov-2 infection and vaccination.

Despite the diversity of individual immune repertoires (1), by position diversity and selection patterns are highly correlated between individuals (2). We and others have hypothesized that this correlation occurs due to the nature of our comparison. When we look at the entirety of immune repertoires that encompasses many individual immune responses, the main localized signature of selection is a negative selection focused on maintaining BCR integrity, which is highly similar across different individuals and responses. To move beyond this bias, we here compared the patterns of selection found in a specific response (SP sub repertoires) to a more general response (SN sub repertoires) within the same individual and between different individuals. By focusing on a specific immune response, we unveiled significant differences in selection patterns between specific anti SARS-CoV-2 responses (SP) and non specific more general responses (SN) (**Figure 1**), that could even be used to differentiate the SP and SN repertoires effectively (**Figure 2**).

Given the hypervariability of the CDR regions (3), our initial intuition was that the reduced correlation would stem from unique patterns originating within these areas. However, to our surprise, the CDR regions, and specifically CDR2 exhibited reduced tolerance toward substitutions in the SP sub repertoires (**Figure 2** and **S2**). This apparent footprint of negative selection suggests that the germline sequence of the CDR2 region may play a key role in maintaining a SARS-CoV-2 response.

Next, we sought to characterize the types of sequences unique to SP sub repertoire. To investigate if such motifs exist, we utilized trimer analysis (overlapping 3-amino acid sequences) (9, 14). This analysis unveiled a germline motif with length of 12 amino acids within the CDR2 region (spanning positions 53 to 66), defined by the sequences ‘VAVISYDGSNKY’ and ‘VAVIWYDGSNKY’, which is constructed from 8 consecutive trimer overlaps (**Figure 6**). The motifs are found only in the germline sequence of IGHV3-30, IGHV3-30-3, IGHV3-30–5 and IGHV3-33 (37, 38).

Our two independent analysis methods - substitution survival and trimer analysis, both converged on the CDR2 region, specifically highlighting the high conservation of the ‘VAVISYDGSNKY’ and ‘VAVIWYDGSNKY’ motifs (**Figures 2**, **7**; **Supplementary Figure 2**), interestingly enough, in previous research about the nature of the interaction between SARS-CoV-2 specific antibodies and the spike RBD site has shown that the ‘SGGS’, found at positions 58–64 (adjusted to IMGT numbering scheme, containing spacers) of the V gene alleles IGHV3–53 and IGHV3-66, serves as a critical binding site to the SARS-CoV-2 RBD (15). This motif contains both serine and glycine at positions 63 and 64 of the heavy chain CDR2, which may hint at the possibility of an RBD binding site. These positions were identified as being under exceptional negative selection (all are in the top quartile for lack of substitutions according to our LPA analysis) and are part of the motif identified regardless of which type of representative sequence we choose to use for our trimer analysis.

Additionally, we quantified selection (31) in the FWR and CDR. Our analysis revealed significant negative selection within the FWR regions, as expected for structural stability. However, we observed a divergence between the CDRs: while CDR1 exhibited positive (diversifying) selection, CDR2 displayed consistent negative selection. These results further affirm our observations that the CDR2 region is highly conserved in this response, while the positive selection in CDR1 highlights the expected hypervariability and affinity maturation characteristic of antigen-driven evolution. The CDR2 conservation we found is supported by research showing that antibodies possessing the IGHV3–30 heavy-chain gene in combination with the IGKV1–39 light-chain gene are frequently observed across different individuals and exhibit high neutralizing potency against SARS-CoV-2 compared to other gene combinations (40). While these previous results strengthen our confidence in our findings it is important to note that they could not on their own single out the CDR2 as the important loci for their selection.

A possible caveat to our identification of selection patterns lies in the existence of structured somatic mutation biases in B cell diversification. These biases target mutations to CDR regions and if unaccounted for can take on the appearance of positive selection in the CDR and negative selection in the FWR (13). However, in general these patterns of mutation should be the same in all sub repertoires and reflected in the domain. Thus, by identifying differences between sub repertoires we should be excluding the effects of somatic mutation and retaining only differences in selection. Furthermore, our specific results in the study of the SP repertoire which exhibits negative selection in the CDR, is not something expected from the somatic mutation biases of B cells which, as stated above, create the impression of positive selection. It has in fact been recently noted that SARS-Cov-2 disease severity can be identified and linked to shifts in the patterns of somatic mutation (4). Cold spots are observed to be significantly more mutable in those suffering from mild disease and hot spots are less mutable in those with severe and mild disease. However, while the first observation is found also when only considering synonymous mutations the latter is only observed when considering all mutations, including non-synonymous ones that could also be affected by (negative) selection (4). This suggests two interesting possibilities – 1) that the observed changes in mutation patterns reflect the heightened dependency on germline CDR2 that we observed here or 2) that our indications of negative selection are a mix of both changes in mutation patterns and selection. Given both the specificity of our observed motif and the basis of the previous results on non-synonymous mutations, the former seems more likely, but in either case we have here pinpointed a region of sequence specificity beyond what was known before. Additionally, our analysis of recovered individuals post vaccination reveals a possibly distinct selection trajectory for vaccine responses. In our PCA analysis of substitution survival the SP sub repertoires spread with each boost. Additionally, we observed a decrease in repertoire correlation (**Figure 1**) and motif conservation (**Figure 7**) over the course of vaccination. This suggests that vaccination may drive distinct SHM patterns that utilize SHM and generate less germline responses which could be more effective or at least complimentary to those evolved during natural infection, under SARS-CoV-2 driven pathological SHM processes.

Beyond these important SARS-CoV-2 specific observations we have also shown here the efficacy of our novel methodology to help us understand the nature of antigen-specific responses. Other methods exist that may identify biases in specific V genes usage (5, 7) and the importance of specific trimer motifs (17, 18). The methodology presented here is not trying to replace them or claim that they are less accurate. What is novel about our method is that it combines a measure of position specific differences in negative and positive selection with the ability to identify over and under-utilization of trimer motifs. In the analysis reported here we showed the impact of more focused negative selection and the importance of a germline-based CDR2 motifs for the anti SARS CoV-2 spike protein response. This conclusion was reached because we could pinpoint the positions where there was a lack of amino acid substations and an overall overabundance of specific trimers. Other responses may involve specific positions where substitutions are over abundant, and the diversity of motifs goes down. Gaining an understanding not just of what amino acid patterns are being selected, but also describing the forces of selection driving their selection is what is novel about the methodology described here. We look forward to having our novel set of metrics applied by us and others to more pathogenic and autoimmune diseases in which both antigen specific and non-specific sub repertoires are observed and to decipher the many different ways the processes of negative and positive selections lead to the formation of specific immune repertoires and responses.

## Data Availability

Publicly available datasets were analyzed in this study. This data can be found here: https://www.ncbi.nlm.nih.gov/bioproject/PRJNA715378.
