# Supplementary material for: Germline based SARS-CoV-2 specific B cell repertoire motif identified with novel sequence based bioinformatic pipeline
Source: Front Immunol. 2026 Aug 5;17:1823750. doi: 10.3389/fimmu.2026.1823750 (PMC13485583; doi:10.3389/fimmu.2026.1823750)
Supplement: Supplementary file 1 [file DataSheet1.pdf]

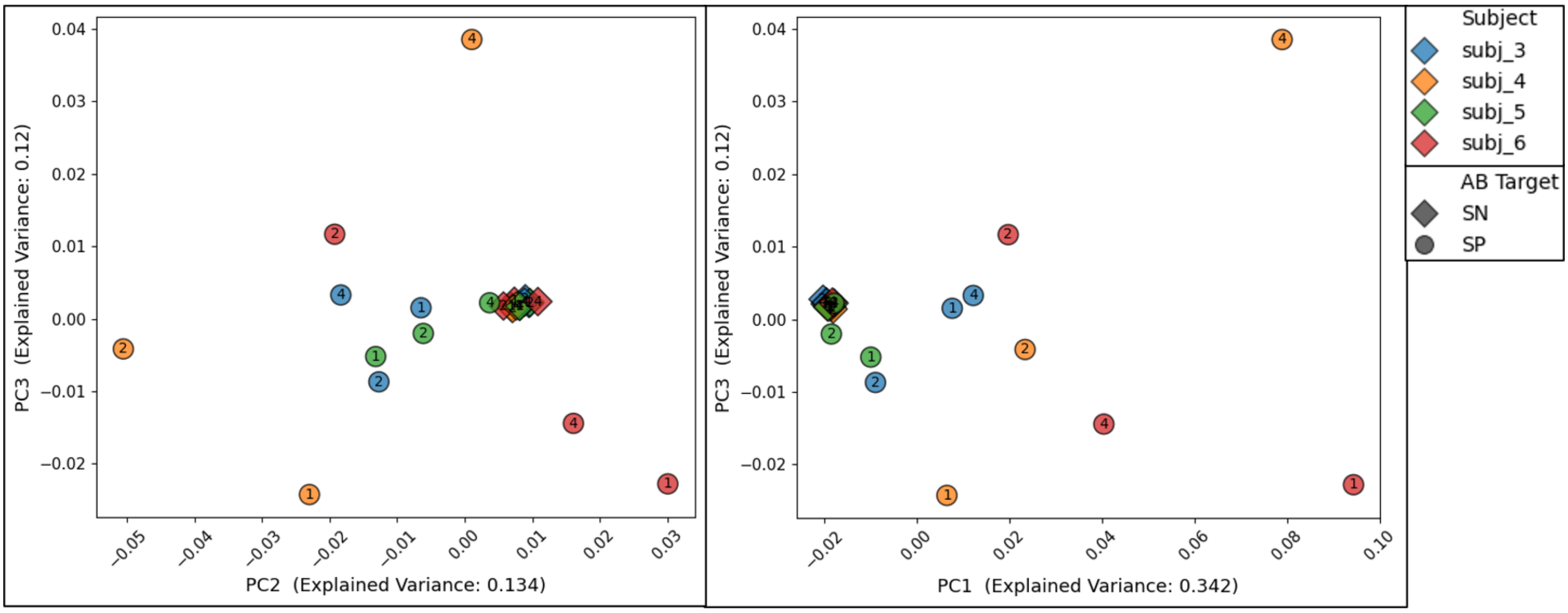

***Figure S1**. Combination of PCA dimensions of repertoire KLDe distances from the domain are based on substitution survival measures. (a)PC1 and PC3 (b) PC2 and PS3. Circles and diamonds represent repertoires with and without SARS-CoV-2 spike protein affinity, respectively. Data points are color-coded by subject, with time points labelled within each symbol (1- pre vaccination, 2-2 weeks before first dose, 4- 3weeks after first dose and 1 week after second dose).*

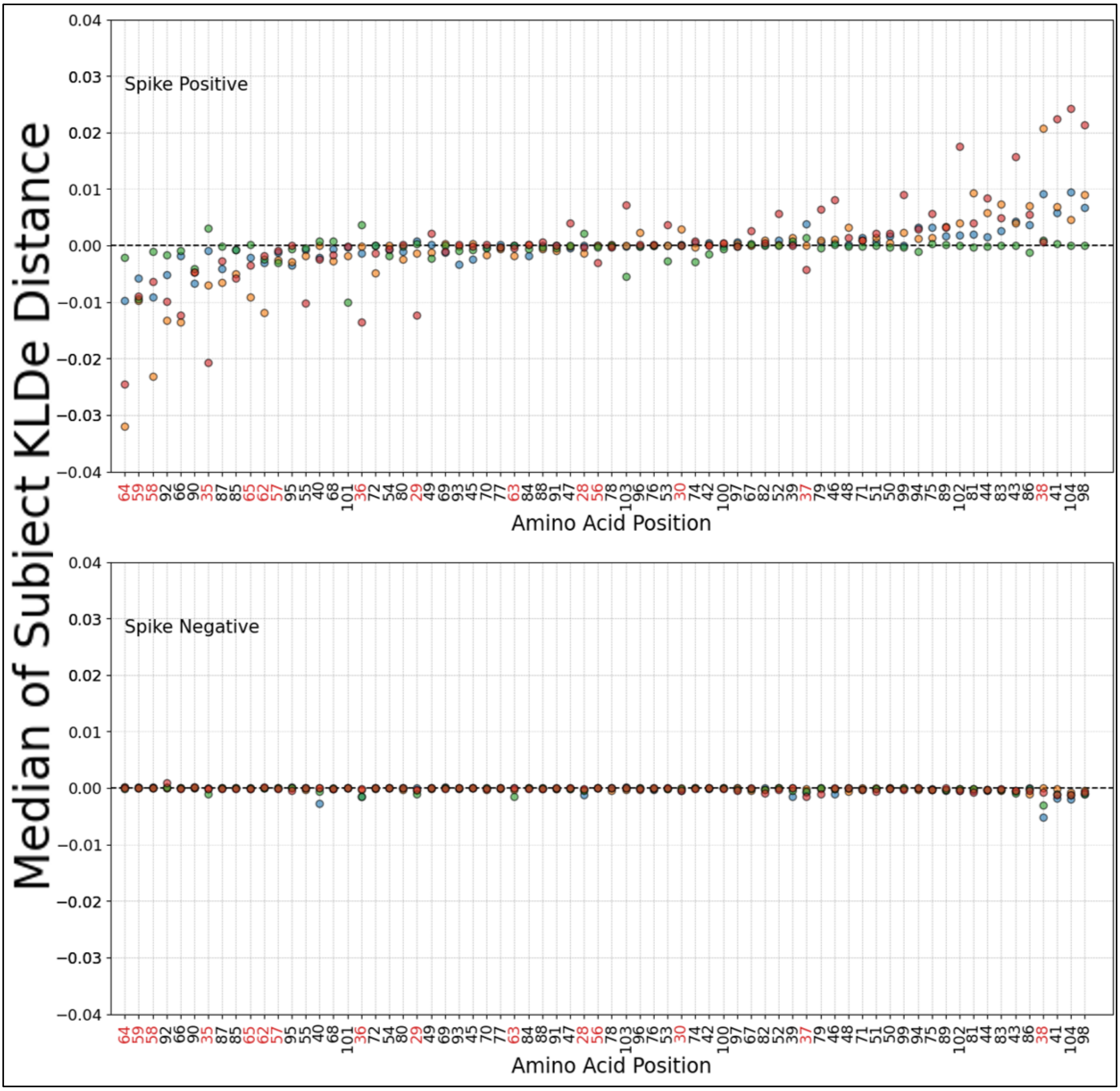

Figure S2. Median KLDe distance of each subject across amino acid positions. Amino acid positions are ordered from highest (right) to lowest (left) median KLDe distance. The top panel displays SP repertoires, while the bottom panel shows SN repertoire. Red position label indicates CDR position. Each subject is marked with different colors: orange (subject 3), green (subject 4), red (subject 5) and purple (subject 6), and blue (subject 7).

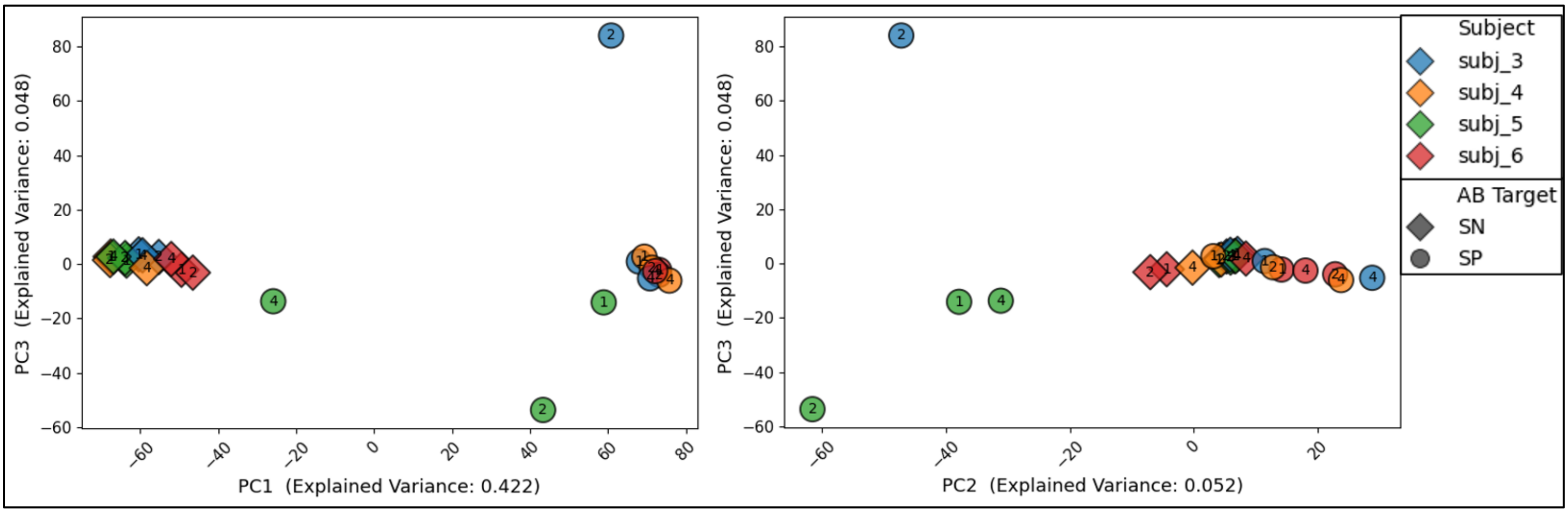

***Figure S3**. Combination of PCA dimensions of repertoire KLDe distances from the domain are based on 3-mers usage frequencies. (a)PC1 and PC3 (b) PC2 and PS3. Circles and diamonds represent repertoires with and without SARS-CoV-2 spike protein affinity, respectively. Data points are color-coded by subject, with time points labelled within each symbol (1- pre vaccination, 2-2 weeks before first dose, 4- 3weeks after first dose and 1 week after second dose).*

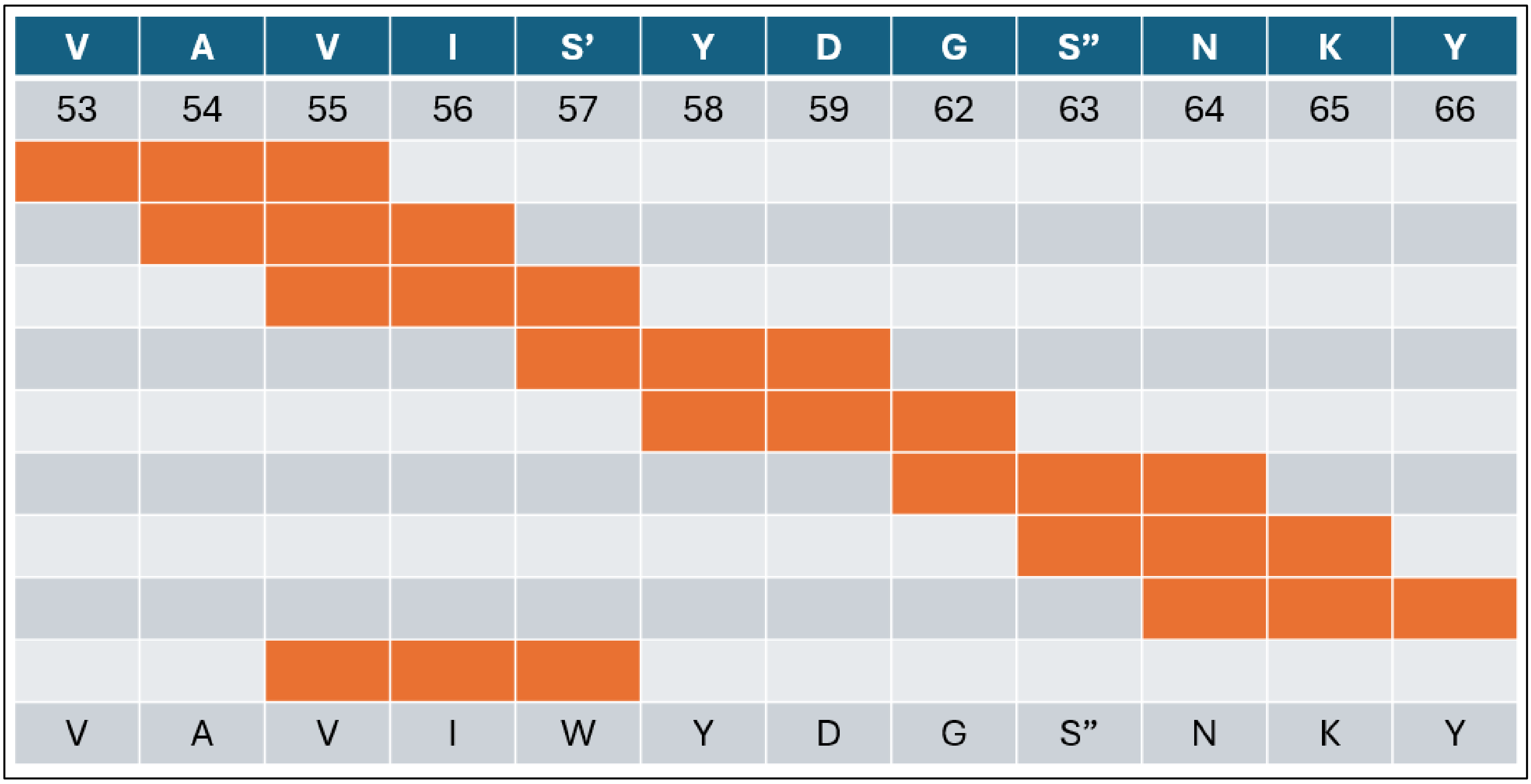

Figure S4. Graphical visualization of the overlapping trimers comprising the motif. Each trimer within the sliding window is indicated by three consecutive orange cells. The primary amino acid sequence and its corresponding positions are shown in the first and second rows, respectively. The bottom row illustrates a motif variant, highlighting the different trimer at positions 55-57.

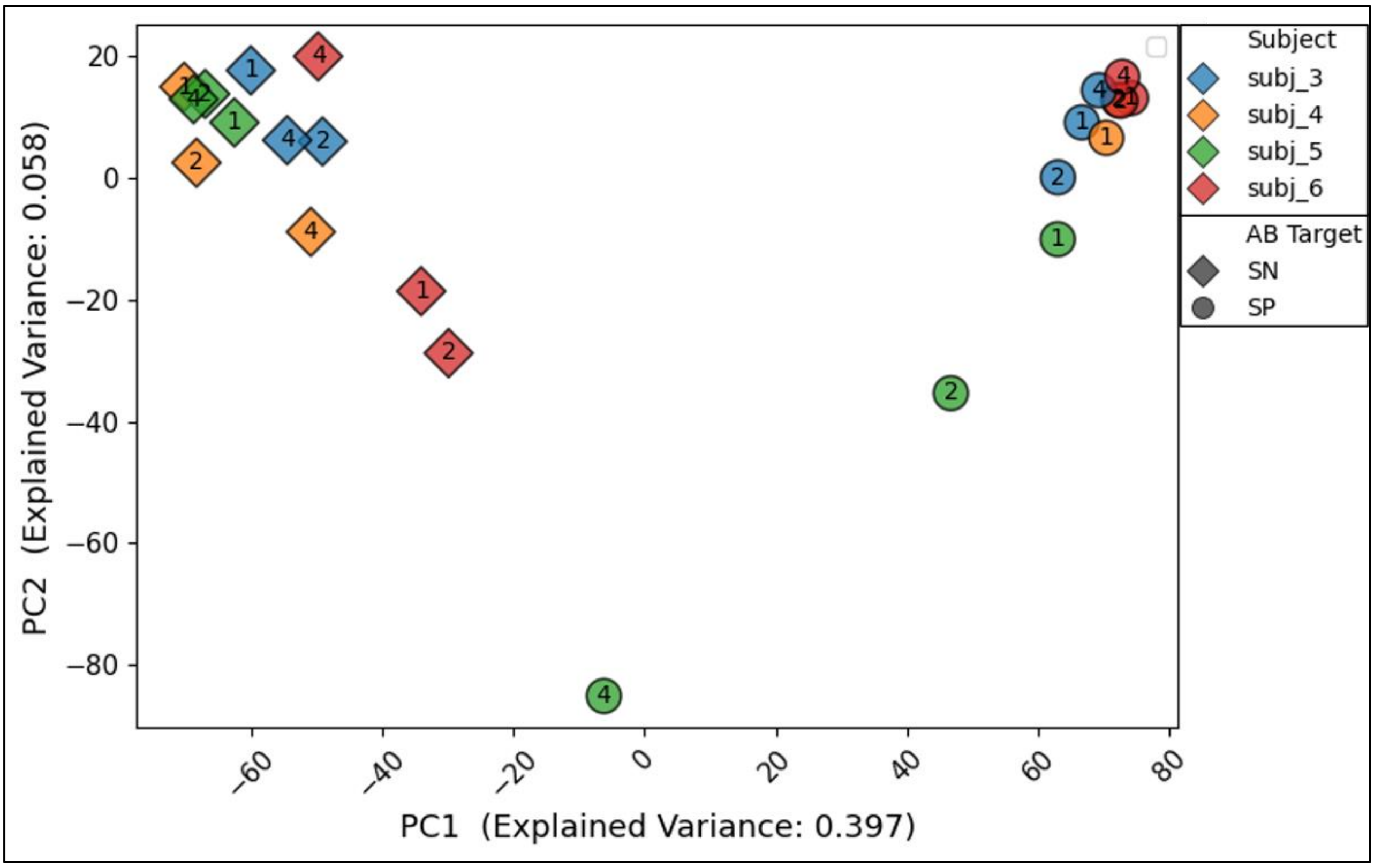

Figure S5. *Scatter plot of PC1 and PC2 combination derived from PCA of repertoire KLDe distances relative to the entire domain which obtained from the trimer occurrences analysis. Circles represent SP sub repertoires and diamonds SN sub repertoires. Data points are color-coded by subject, as in Figure 3, with time points labelled within each symbol (1- pre vaccination, 2-2 weeks before first dose, 4- 3weeks after first dose and 1 week after second dose). This analysis represents each clone by its consensus sequence (see Methods) rather than the most abundant sequence to represent each clone.*

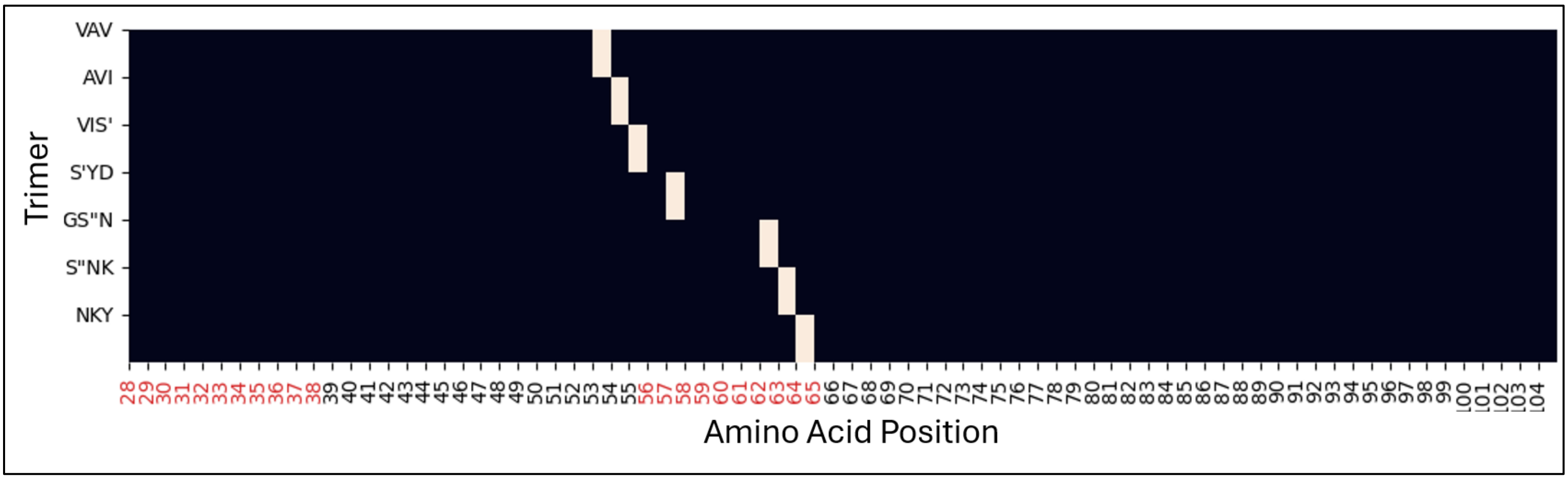

Figure S6. *Heatmap of unique occurrences overabundant in SP repertoires based on KLDe domain distance median score per individual and organized by the s' first IMGT heavy chain sequence position. CDR positions are highlighted in red. Only s overabundant in at least 2 individuals (in the top quartile of overabundance) were noted. Cell color reflects prevalence: beige-white color denotes presence in 4 individuals, while black indicates total absence. This analysis represents each clone by its consensus sequence (see* ***Methods****) rather than the most abundant sequence to represent each clone.*

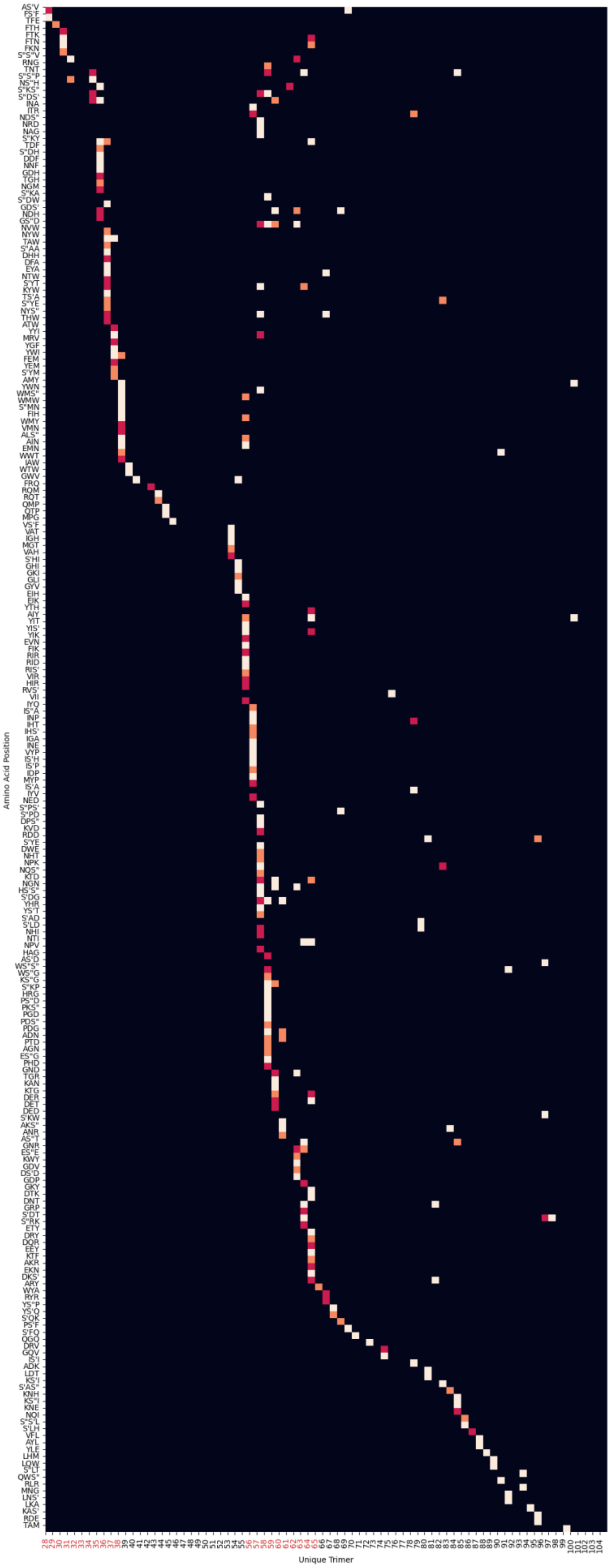

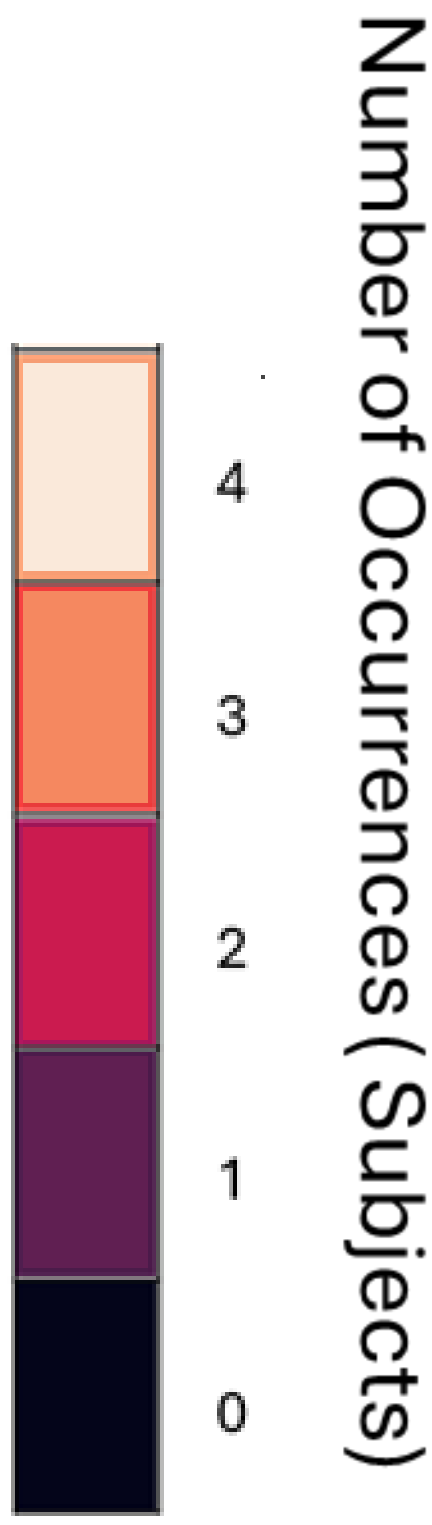

Figure S7. *Heatmap of unique 3-mer occurrences overabundant in SN repertoires based on KLDe domain distance median score per individual and organized by the 3-mers' first IMGT heavy chain sequence position. CDR positions are highlighted in red. Only 3-mers overabundant in at least 2 individuals (in the top quartile of overabundance) were noted. The color intensity indicates the number of subjects in which the 3-mer was found from beige white (all of the subjects) to black (none), as presented in the legend.*

Table S1. Total, SP and SN unique clones count per subject.

Subject ID	Unique Clones Count (Total)	Unique SN Clones	Unique SP Clones
3	13039	11476	1563
4	19433	18702	731
5	21024	17373	3651
6	9398	8771	627
7	5713	5544	169
